# Communicating mild cognitive impairment diagnoses with and without amyloid imaging

**DOI:** 10.1186/s13195-017-0261-y

**Published:** 2017-05-04

**Authors:** Joshua D. Grill, Liana G. Apostolova, Szofia Bullain, Jeffrey M. Burns, Chelsea G. Cox, Malcolm Dick, Dean Hartley, Claudia Kawas, Sarah Kremen, Jennifer Lingler, Oscar L. Lopez, Mark Mapstone, Aimee Pierce, Gil Rabinovici, J. Scott Roberts, Seyed Ahmad Sajjadi, Edmond Teng, Jason Karlawish

**Affiliations:** 1 Institute for Memory Impairments and Neurological Disorders, University of California, Irvine, CA USA; 2 Department of Psychiatry and Human Behavior, University of California, Irvine, CA USA; 3 Department of Neurology, University of California, Irvine, CA USA; 4 Alzheimer’s Disease Center, Department of Neurology, Radiology, Medical and Molecular Genetics, University of Indiana, Indianapolis, IN USA; 5 University of Kansas, Kansas City, KS USA; 6 Alzheimer’s Association, Chicago, IL USA; 7 Mary S. Easton Center for Alzheimer’s Disease Research, Department of Neurology, David Geffen School of Medicine at University of California, Los Angeles, CA USA; 8 University of Pittsburgh, Pittsburgh, PA USA; 9 University of California, San Francisco, CA USA; 10 University of Michigan School of Public Health, Ann Arbor, MI USA; 11 University of Pennsylvania, Philadelphia, PA USA; 12 Veterans Affairs Greater Los Angeles Healthcare System, Los Angeles, CA USA; 13 Institute for Memory Impairments and Neurological Disorders, University of California, 3204 Biological Sciences III, Irvine, CA 92697 USA

**Keywords:** Mild cognitive impairment, Diagnosis, Disclosure, Prognosis, Amyloid imaging

## Abstract

**Background:**

Mild cognitive impairment (MCI) has an uncertain etiology and prognosis and may be challenging for clinicians to discuss with patients and families. Amyloid imaging may aid specialists in determining MCI etiology and prognosis, but creates novel challenges related to disease labeling.

**Methods:**

We convened a workgroup to formulate recommendations for clinicians providing care to MCI patients.

**Results:**

Clinicians should use the MCI diagnosis to validate patient and family concerns and educate them that the patient’s cognitive impairment is not normal for his or her age and education level. The MCI diagnosis should not be used to avoid delivering a diagnosis of dementia. For patients who meet Appropriate Use Criteria after standard-of-care clinical workup, amyloid imaging may position specialists to offer more information about etiology and prognosis. Clinicians must set appropriate expectations, including ensuring that patients and families understand the limitations of amyloid imaging. Communication of negative results should include that patients remain at elevated risk for dementia and that negative scans do not indicate a specific diagnosis or signify brain health. Positive amyloid imaging results should elicit further monitoring and conversations about appropriate advance planning. Clinicians should offer written summaries, including referral to appropriate social services.

**Conclusions:**

In patients with MCI, there is a need to devote considerable time and attention to patient education and shared decision-making. Amyloid imaging may be a tool to aid clinicians. Careful management of patient expectations and communication of scan results will be critical to the appropriate use of amyloid imaging information.

## Background

Mild cognitive impairment (MCI) is defined as lower cognitive performance than expected for a person’s age and education that elicits complaint, does not affect activities of daily living, and does not meet criteria for dementia [1]. Professional organizations and expert workgroups have developed diagnostic criteria for MCI [2–4] and the construct is included in the 10th revision of the International Statistical Classification of Diseases and Related Health Problems (G31.84) and the fifth edition of the *Diagnostic and Statistical Manual of Mental Disorders* (as mild neurocognitive disorder).

Primary care physicians and dementia specialists use the MCI diagnosis to help patients and families understand patient symptoms, motivate planning, and spur risk-reducing behaviors. Yet the construct remains controversial. One source of this controversy is the value of the label [5, 6]. Persons with MCI progress to dementia at a rate of 12–16% per year [7]. Long-term studies, however, show that a significant proportion of MCI patients do not progress to dementia and a subset may revert to normal cognition [8, 9]. These data foster criticism of the MCI diagnosis as enmeshed with ambiguity and uncertainty and therefore a cause of unnecessary worry [6]. Clinicians may in turn be challenged by how to discuss MCI with their patients.

The US Food and Drug Administration’s recent approval of three amyloid-specific positron emission tomography (PET) ligands [10–12] may affect these controversies and challenges. Amyloid PET imaging provides clinicians with additional information about the cause of a patient’s MCI. A positive amyloid PET result is highly predictive of the presence of fibrillar amyloid-beta pathology, a hallmark of Alzheimer’s disease (AD) [13]. Among those with MCI, elevated amyloid conveys increased risk for progression to dementia [14, 15]. Risk is highest if evidence of both amyloidosis and neurodegeneration are present [16, 17]. Although most MCI patients with elevated amyloid also demonstrate markers of neurodegeneration [18], the risk for progression to dementia in those with elevated amyloid who do not demonstrate neurodegeneration may be no greater than the risk in MCI patients with evidence of neurodegeneration alone [16–18]. Positive amyloid PET can also occur in other brain diseases that involve fibrillar amyloid deposition, such as dementia with Lewy bodies or cerebral amyloid angiopathy [19], while a portion of persons labeled with AD dementia lack elevated amyloid [20]. Elevated amyloid is also present in approximately 25% of cognitively normal older individuals, with higher prevalence found with increasing age and in carriers of the apolipoprotein E ε4 allele [21]. Therefore, positive amyloid PET is not equivalent to AD diagnosis and the meaning of amyloid PET results in a person with MCI must be interpreted in the context of a patient’s entire clinical assessment, including medical history, cognitive testing, and structural neuroimaging.

An essential value of the MCI construct is that it provides information about prognosis, and the manner in which the MCI diagnosis is delivered could substantially alter the clinical interaction [22]. Amyloid imaging provides the ability to label a person with a pathology seen in AD dementia, a disease that is the cause of substantial social, cultural, and ethical anxieties and concerns. We conducted a workgroup meeting to identify recommendations and best practices for delivering the MCI diagnosis, with and without the availability of amyloid imaging information, and to outline areas in need of research related to these topics.

## Methods

A workgroup meeting was funded by a “ChangeAGEnts” grant from the American Federation for Aging Research and the Hartford Foundation. The funding application outlined the meeting agenda, participants, and topics for discussion. The agenda was further developed through teleconferences among a subset of participants prior to the in-person meeting.

The in-person meeting was held on February 19, 2016 at the Institute for Memory Impairments and Neurological Disorders on the campus of the University of California, Irvine, USA. A list of the meeting attendees can be found in the Acknowledgements section. The agenda included presentations on MCI and amyloid imaging and discussion of the following topics: the value of the MCI diagnosis; the role of family members and other people in making the MCI diagnosis; how to discuss the MCI diagnosis; and what information should be provided at the MCI diagnosis.

At the conclusion of the workgroup meeting, one author (JDG) used the agenda, presented lectures, and meeting notes to draft a summary of the discussion, the proposed recommendations, and areas of needed study. This draft was shared with all authors for revision and discussion. The final product of this process is represented in the current manuscript.

## Recommendations

The workgroup discussed delivering an MCI diagnosis in the context of the current state of science and practice. As disease-modifying therapies become available, the clinical interaction in MCI and the use of amyloid PET are certain to change. Unless these therapies effectively prevent the onset of MCI, the importance of how to communicate an MCI diagnosis with or without biomarker information will endure. The term AD has been used to describe two distinct entities: a clinical syndrome (e.g., dementia) and a specific pathophysiology (e.g., the presence of amyloid plaques and neurofibrillary tangles). Here, we use the pathophysiological definition of AD but limit the use of this term to patients with demonstrated cognitive impairment. While it is clear that other biomarkers can provide information similar to that provided by amyloid imaging (e.g., cerebrospinal fluid (CSF)), our group considered amyloid PET specifically, because this test is one of few Food and Drug Administration-approved and commercially available AD biomarkers. Furthermore, the Centers for Medicare and Medicaid Services are supporting Imaging Dementia Evidence for Amyloid Scanning, a study that will test the value of amyloid imaging in clinical practice (http://www.ideas-study.org/). An Amyloid Imaging Task Force developed Appropriate Use Criteria that reserve prescribing and disclosing amyloid PET results for dementia specialists, defined as clinicians who devote at least 25% of their patient contact time to the evaluation and care of adults with acquired cognitive impairment or dementia [23, 24]. Yet most MCI patients will not receive specialist care. Therefore, any guidance must recognize the wide range of practitioners who care for MCI patients. Some of our recommendations are therefore limited to dementia specialists (e.g., those pertaining to amyloid imaging), and other recommendations are applicable to both dementia specialists and general practitioners who see patients with memory complaints (e.g., those pertaining to general MCI diagnosis).

### Recommendation 1 – workup, with standard laboratory tests, neuropsychological assessment, and structural brain imaging, is required to arrive at the diagnosis of MCI. The MCI diagnosis validates patient and family concerns and facilitates planning. The MCI diagnosis should not be used to avoid delivering a diagnosis of dementia

Clinicians who see cognitively impaired patients have incentives and disincentives to make the MCI diagnosis. Incentives include ensuring patient safety and assisting in planning related to the diagnosis (e.g., patients living alone, the need for support, management of financial affairs), validating patients’ and families’ concerns, considering treatment options, and referring patients to clinical trials and other research studies. Disincentives include affecting a person’s access to insurance and employment and other issues related to discrimination and stigma. Comprehensive neuropsychological assessment provides clinical detail that is not provided by brief global assessments that are typically performed in a physician’s practice, but is limited by the availability of clinical neuropsychologists. The time required to properly diagnose MCI, including the need for patient and family education and counseling, may also be a disincentive to some clinicians.

Given the safety implications for driving and finances in mild-stage dementia, as well as in some US states the requirement to report dementia to driving authorities [25], the MCI diagnosis should not be given to patients with cognitive impairment that affects activities of daily living. Similarly, the diagnosis should not be used to avoid discussing the possibility of underlying neurodegenerative disease, if the clinical workup supports such an etiology.

### Recommendation 2 – patients should have an informant present to assist in the diagnostic process. The preference of MCI patients who decline bringing an informant should be respected, but they should understand that this preference limits the information needed for the diagnostic process and patient care

Having an informant present for diagnostic assessment and disclosure of an MCI diagnosis is optimal. Family members provide valuable collateral information, especially about whether a patient’s cognitive performance is causing functional impairment. Family members also provide social and emotional support and can ensure that clinical recommendations are followed. Our group agreed that clinicians should strongly recommend that patients involve others in the process, but we recognized that some patients prioritize privacy and confidentiality and prefer to be evaluated alone. We concluded that after a discussion of the benefits of including family, the patient’s personal preferences must take precedence. Including family members may also create challenges. They may disagree over decisions about the diagnostic workup. Our group felt that the clinician should encourage consensus, but if consensus cannot be achieved then the patient’s wishes should govern these decisions. If the decision involves amyloid imaging, this requires the clinician to assess that the patient adequately understands the role of amyloid imaging in the MCI workup.

### Recommendation 3 – clinicians must carefully set expectations at the start of the workup of patients with cognitive complaints. This should include the need to rule out treatable causes of impairment and a determination of patients’ desire to learn more about the cause of their memory loss

When initiating a diagnostic workup for cognitive complaints, clinicians need to set expectations. This includes communicating the need to assess for medically treatable causes of impairment and safety. Beyond these standard elements, the clinician should assess the patient’s and family’s desire to pursue additional etiologic testing, recognizing that it is a highly individualized decision and that both MCI and AD dementia carry stigma.

### Recommendation 4 – the MCI diagnosis should be used as an opportunity to help patients validate and understand their condition. MCI should be described as cognitive performance that is below that expected for patients’ age and education

The diagnosis of MCI is an opportunity for the clinician to validate the subjective complaints that brought the patient to the clinic. If the assessment reveals cognitive impairment, the clinician should communicate that the patient’s performance is abnormal relative to expectations for their age and education. Validation of the patient’s complaints (“My assessment confirms that you do have memory performance below what we expect for someone your age”) and reassurance are important to communicate to the patient.

The delivery of MCI diagnosis requires careful, honest, and compassionate dialog. Table 1 outlines example language that might be used to deliver the MCI diagnosis.

**Table 1:** Example language to communicate the MCI diagnosis

Patient description	Example language to deliver diagnosis
MCI believed to be caused by AD	“Your complaints are concerning and not what we expect for a person your age. Any time someone presents with these types of memory concerns later in life, I worry that it is the early stages of Alzheimer’s disease.” “Although we can’t predict outcomes on an individual basis, people with cognitive performance similar to yours are at increased risk for Alzheimer’s dementia. We’re going to watch you closely and do everything we can to help your memory performance and lower your risk for future decline.”
MCI believed to be caused by a non-AD neurodegenerative condition or uncertain etiology	“I’m concerned that the types of changes in cognitive performance you are experiencing suggest the possibility of brain disease. There are some things we can discuss to try to help you with these symptoms, but we may need to run some more tests to try to determine what is causing these changes if that is something you wish to pursue.”
MCI with positive amyloid PET	“Your scan results suggest that amyloid levels in your brain are elevated. Combined with the other tests we’ve done, it leads me to conclude that Alzheimer’s disease is the most likely cause of your cognitive changes, although other less likely causes remain a possibility. Although I can’t be absolutely certain and we don’t have the individual estimates for timing, people with results like yours are at increased risk for developing dementia over the next few years. Given all of this, I think we need to talk about making an overall plan to manage your condition.”
MCI with negative amyloid PET	“Your scan results did not indicate that there is a significant amyloid burden at this time. This suggests that Alzheimer’s disease pathology is not currently present in your brain and your risk for getting dementia is lower than had the scan found amyloid build up. The scan results could change in the future but this could also mean that another brain disorder may be causing your cognitive changes. We still need to try to figure out why you are having these symptoms. We will do that together.”

*AD* Alzheimer’s disease, *MCI* mild cognitive impairment, *PET* positron emission tomography

### Recommendation 5 – clinicians should consider reviewing patients’ performance on tests of specific cognitive domains when delivering the MCI diagnosis. This review should include cognitive domains that demonstrate impaired performance (and support the diagnosis of MCI) and the domains that demonstrate unimpaired performance

Our group concluded that a patient’s understanding of their cognitive complaints could be facilitated by reviewing their cognitive testing results, including the specific cognitive domains in which test results are “below what we expect for a person’s age and education” and the domains in which test results are within normal ranges or above average. Not all patients will be interested in this information, but those who are may benefit from such discussions.

A review of cognitive domains is facilitated by referral to a clinical neuropsychologist. Impairment in multiple cognitive domains including memory is associated with increased risk for progression to dementia, compared with single domain amnestic or multiple domain nonamnestic presentations. Moreover, greater severity of impairment is associated with greater risk for progression to dementia [26].

If appropriate, reviewing structural neuroimaging results may also be helpful for patients and families.

### Recommendation 6 – patients should be provided with a written summary of the diagnosis and treatment recommendations that includes referral to appropriate supportive services and other local resources

Written [27] and pictographic [28] information can facilitate learning and understanding. Although our group stopped short of recommending pictorial information to illustrate the risk of dementia among MCI patients, all acknowledged that it could facilitate the clinical interaction. Advocacy organizations, such as the Alzheimer’s Association, could develop informational pamphlets and other materials in partnership with experts, which could be disseminated and used by clinicians providing care to patients with cognitive impairment. Written information about the diagnosis, including referrals to supportive services, is essential.

### Recommendation 7 – MCI patients who are appropriate for amyloid imaging should be offered the opportunity to discuss having the scan, the possible results, and the implications for prognosis, well-being, and management

Patient and family responses to receiving the MCI diagnosis range from relief related to not receiving an AD diagnosis to distress about the risk of dementia [29]. The ambiguity and uncertainty of the diagnosis [30] cause many patients to desire more information about their condition, such as how it differs from “normal” aging and how it differs from dementia [31]. Amyloid imaging may allow a physician to give the patient additional information about potential causes of their MCI, improve prognostic information, and reduce the ambiguity and uncertainty associated with the diagnosis [29, 30, 32–34]. Although some patients may want this information, others may express a desire “not to know” [35]. Discussion is necessary to help patients and families decide whether to have the scan and to set expectations about how the results will affect clinical management [36].

### Recommendation 8 – if an amyloid scan is considered useful by both the clinician and the patient, clinicians should carefully set patients’ and families’ expectations before ordering amyloid imaging. This includes education about the possible scan results, implications, limitations, and cost. Amyloid scans should be described as a test to aid the clinician in creating a more complete understanding of the cause of patients’ MCI

A clinician must carefully set patients’ and families’ expectations before ordering amyloid imaging. This should include the possible results of the scan and their implications, including that information related to only one of the two pathologies needed for the diagnosis of AD will be known. Clinicians must explain that the scan alone will not provide a diagnosis of AD, and it will not provide information regarding the amount of amyloid. The clinician should also explain that the scan does not provide information about disease progression. Patients and families should be encouraged to envision how they would think and feel in the event of positive and negative scan results. This dialog will assist the patient and family, as well as the clinician, to decide whether to undergo the scan. A publication generated by amyloid imaging experts and the Alzheimer’s Association may provide a useful tool to facilitate setting patient and family expectations [37]. Because at the present time amyloid scans are reimbursed only in specific research contexts, providers should also discuss with patients and families the cost of the scan.

### Recommendation 9 – the delivery of amyloid imaging results should occur at an in-person encounter and should be described using terminology related to the presence or absence of amyloid

Food and Drug Administration-approved indications for the available amyloid PET ligands incorporate dichotomous (positive versus negative) results. Different terms such as “elevated” versus “not elevated” and amyloid “build up” versus “no significant build up” have been used in the research setting to avoid confusion, given that a “positive” scan has negative implications for health (Table 1) [38, 39]. While this variable and changing language may introduce additional questions and the desire for quantitation of amyloid burden “severity,” our group concluded that insufficient evidence is available to support providing patients with standardized uptake value ratios or other quantitative estimates, and that doing so could inappropriately suggest a link to disease severity.

### Recommendation 10 – communication of negative scan results should include that patients with MCI and a negative scan remain at risk for dementia and that negative scans, while informative, do not indicate a specific diagnosis or unambiguously signify the absence of disease

Negative results reduce the likelihood of AD as the cause of MCI, as well as the probability of progression to dementia at 2 and 5 years [40, 41]. A negative scan, however, does not absolutely rule out that the patient will progress to clinical AD dementia; neither does it mean that the patient will not progress to dementia caused by another neurodegenerative disease [15].

### Recommendation 11 – positive amyloid PET scan results in patients with MCI are associated with increased risk for developing AD dementia. It is important to discuss the risk for cognitive and functional decline and the need for additional monitoring and planning in these patients

Positive scans are associated with increased risk for progression to AD dementia [41]. Longer follow-up appears to improve the value of positive amyloid PET for predicting decline or conversion to dementia [17, 18, 41]. Amyloid PET alone cannot predict the trajectory of an individual patient’s cognitive decline or the time to progression to any specific outcome. Additional clinical information, such as volumetric brain imaging and detailed cognitive testing, may further assist the clinician in assessing overall prognosis. Patients with positive amyloid scans and evidence of neurodegeneration may be at greatest risk for near-term decline [16]. Regardless of the clinical presentation, including amyloid PET results, uncertainty will remain in assessing prognosis.

### Recommendation 12 – clinicians who are trained to read amyloid scans and to describe the results to patients might consider reviewing images with patients and families

Understanding neuroimaging results can be challenging for patients and families, and preliminary data suggest that showing patients their amyloid scans and comparing them with a scan with the opposite result may facilitate the clinical interaction [34]. Clinicians may consider offering to review these images with patients and their families, but only if they are skilled in reading and explaining the scans and answering questions about the images.

## Areas of need

Our group agreed that there remain several areas in need of further evaluation or research related to the disclosure of MCI diagnosis.

A group composed of dementia experts, with a preponderance of clinical neurologists, formulated these recommendations. Other specialists or general practitioners might arrive at alternative recommendations. Additional meetings could identify these differences and aid in the development of more universal recommendations for communicating an MCI diagnosis. Similarly, we did not include MCI patients or family members in our meeting and their perspectives will be critical to optimizing recommendations.

These recommendations are based on a clinical interaction in which the clinician has the opportunity to provide adequate education and counseling. They may be less practical for busy general practitioners for whom limited time is available but the MCI diagnosis remains frequent and important.

More work is needed on the topic of returning quantitative amyloid PET results to patients. Image quantification is not currently part of clinical practice and is performed primarily in the research arena. Patients may wish to learn their quantitative results and clinicians must be prepared for this discussion. Evidence is lacking to instruct the implications of greater versus lesser amyloid burden. In contrast, more severe cognitive impairment is related to greater risk for progression to dementia [26] and preliminary studies suggest that cognitive measures are as predictive of progression to dementia as are biomarkers [42, 43]. Whether and how cognitive impairment severity and amyloid burden severity may relate to instruct prognostication is unknown. Amyloid imaging results strongly correlate with amyloid levels in CSF [44], and these recommendations may largely apply to disclosing results of that biomarker test. CSF, however, provides additional information that may facilitate adjustment of these recommendations [45]. Furthermore, the inclusion of other biomarkers such as regional glucose metabolism, brain volume, or information on tau pathology will increase the information for clinicians to use in assessing prognosis. Ultimately, risk curves that incorporate amyloid imaging outcomes, cognitive scores, and other relevant clinical and biomarker information may optimize discussion of prognosis. These remain areas of active study.

Finally, these recommendations can and should be tested to show their short-term and long-term effectiveness of increasing patient understanding, affecting health behaviors, and improving the clinical interaction.

## Conclusions

MCI is a common diagnosis that raises important safety issues, is associated with uncertainty about etiology and prognosis, and can result in anxiety for patients and families. The inappropriate or inadequate delivery of the MCI diagnosis could carry substantial risks for patients and families. Language selected to deliver this diagnosis is key to optimizing the clinical encounter and ensuring patient understanding, health behavior outcomes, and patient safety. Amyloid PET imaging adds to the information available to clinicians caring for MCI patients and may enable more confident discussion of disease etiology, treatment, and potential clinical trajectories [46–51].

There is a need to devote considerable time and attention to ensuring patient desires and understanding, especially before engaging in diagnostic testing to elucidate disease etiology. In patients who seek information, amyloid imaging can be a valuable tool to assess disease pathology and to aid the clinician in counseling and treatment recommendations. Careful setting of expectations and delivery of scan results will be critical to the appropriate use of amyloid imaging information that enhances the clinical interaction.

## Abbreviations

AD: Alzheimer’s disease
MCI: Mild cognitive impairment
PET: Positron emission tomography
